# On‐Demand Sintering of Gold Nanoparticles via Controlled Removal of o‐Nitrobenzyl Thiol Ligands Under Record‐Low Power for Conductive Patterns

**DOI:** 10.1002/advs.202415496

**Published:** 2025-01-31

**Authors:** Jisun Im, Charles Heaton, Nur R. E. Putri, Changxu Liu, Junichi Usuba, Kevin Butler, Michael Fay, Grace G. D. Han, Helia Hooshmand, Adam Thompson, Ricky Wildman, Richard Hague, Lyudmila Turyanska, Christopher Tuck

**Affiliations:** ^1^ Centre for Additive Manufacturing Faculty of Engineering University of Nottingham Nottingham NG7 2RD UK; ^2^ School of Engineering University of Warwick Coventry CV4 7AL UK; ^3^ Centre for Metamaterials Research and Innovation Department of Engineering University of Exeter Exeter EX4 4PY UK; ^4^ Research Center for Net Zero Carbon Society Institute of Innovation for Future Society Nagoya University Nagoya Japan; ^5^ School of Chemistry University of Nottingham Nottingham NG7 2RD UK; ^6^ Nanoscale and Microscale Research Centre University of Nottingham Nottingham NG7 2RD UK; ^7^ Department of Chemistry Brandeis University Waltham MA 02453 USA; ^8^ Manufacturing Metrology Team Faculty of Engineering University of Nottingham Nottingham NG7 2RD UK

**Keywords:** 3D printing, additive manufacturing, conductive inks, metal nanoparticles, photocleavable ligands, photonic sintering

## Abstract

Metal nanoparticles‐based nanoinks have shown potential for fabricating metallic components essential to the realization of innovative 3D‐printed electronic devices. However, fabricating metallic patterns on flexible, heat‐sensitive substrates remains challenging due to high temperature and high energy sources, such as intense pulsed light (IPL), involved in the sintering process. Here an efficient sintering method is presented using ultralow power UV by leveraging the photocleavable ligand, *o*‐nitrobenzyl thiol (NT), – functionalized gold nanoparticles (AuNPs). The controlled removal of NT ligands upon UV irradiation enhances light absorption by reducing the filling factor of voids in the printed layer, increasing the layer temperature, and facilitating further ligand desorption. This positive feedback mechanism accelerates nanoparticle sintering at several orders of magnitude lower energy than IPL, achieving an electrical conductivity of 7.0 × 10^6^ S m^−1^. This nanoink promises the parallel printing of multimaterial components through ultralow power photonic sintering for fabricating multifunctional 3D‐printed electronic devices.

## Graphical abstract

An innovative strategy to fabricate 3D‐printed metallic patterns on flexible substrates is developed by leveraging photocleavable ligand‐functionalized metal nanoparticles. The nanoink undergoes ligand cleavage upon UV irradiation, facilitating particle coalescence and fusion. This method achieves the metallic conductivity of the inkjet‐printed gold patterns with record‐low UV power for photonic sintering, enabling flexible electronics production.

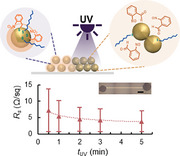

## 1 Introduction

3D printed electronic components with high levels of geometric complexity open up numerous opportunities, not only for integrated electronic circuits^[^ ^1^ ^]^ but also for innovative applications such as flexible, wearable electronics,^[^ ^2^, ^3^ ^]^ wireless communications,^[^ ^4^ ^]^ energy generation and storage devices,^[^ ^5^, ^6^ ^]^ biomedical devices,^[^ ^7^, ^8^ ^]^ and other complex sensor/actuator systems.^[^ ^9^, ^10^ ^]^ Thus, additively manufacturable conductive materials are of great interest for fabricating hierarchical complex electronic systems and multifunctional 3D printed electronic device integration. Nanoinks containing metal nanoparticles (MeNPs) such as gold (Au),^[^ ^11^, ^12^ ^]^ silver (Ag),^[^ ^13^, ^14^ ^]^ and copper (Cu)^[^ ^15^, ^16^ ^]^ have proven effective for fabricating printed electronic components. Their easily controllable particle sizes and surface functionalities allow for the adjustment of ink stability, printability, and postprocessing. As‐printed MeNPs in the quantum size regime (less than 2 nm in diameter) are insulating due to discrete electron energy levels and the organic ligand shells surrounding the MeNPs.^[^ ^17^ ^]^ However, metallic conductivity of the printed MeNPs can be achieved by creating continuous conductive pathways through particle coalescence and fusion during the sintering process.^[^ ^11^, ^12^, ^13^, ^14^, ^15^, ^16^ ^]^

Thermal sintering, a common process for MeNPs‐based nanoinks, involves surface ligand desorption and particle fusion at temperatures much lower than the bulk melting point (T_m_), due to the significant decrease in T_m_ with smaller particle sizes. Thermally sintered MeNPs printed patterns can achieve electrical conductivities of 10^6^ to 10^7^ S m^−1^ at a sintering temperature between 130 to 300 °C for 30–60 min.^[^ ^18^, ^19^ ^]^ Although thermal sintering is effective and widely adopted, it can deform flexible, heat‐sensitive polymer substrates with lower glass transition temperatures (T_g_), causing cracks and delamination in printed structures due to sintering temperatures exceeding T_g_.

Photonic sintering, including laser sintering using a focused laser beam and intense pulsed light (IPL) sintering using a xenon flash lamp, has recently gained great interest due to its short processing time (often less than a second) and compatibility with roll‐to‐roll manufacturing processes.^[^ ^20^, ^21^ ^]^ Both laser and IPL sintering methods demonstrate efficacy in achieving electrical conductivity comparable to thermal sintering.^[^ ^22^, ^23^, ^24^, ^25^, ^26^, ^27^, ^28^, ^29^, ^30^ ^]^ However, they involve high energy processes requiring peak power densities of 10^2^–10^5^ W cm^−2^ to reach peak temperatures of 300 to 600 °C. Excessive pulse energy in IPL sintering can cause defects such as morphological variations, cracks, delamination, inhomogeneous shrinkage in the metal film, and damage to the substrate.^[^ ^22^, ^27^, ^31^, ^32^ ^]^ To mitigate these issues, pulsed sintering parameters such as energy, pulse duration, peak power, and number of pulses must be carefully controlled and optimized for different inks and substrates to ensure uniform sintering.^[^ ^33^ ^]^ Therefore, developing a low‐energy photonic sintering method compatible with scalable, cost‐effective manufacturing and low‐energy light sources is crucial, especially for applications such as flexible electronics that require high precision, low power, and minimal thermal impact. In addition, ultralow‐energy sintering can enable the integration of heat‐sensitive components, opening possibilities for multimaterial 3D printing.

In this work, we unveil an innovative strategy for fabricating conductive patterns via photonic sintering of nanoinks using ultralow‐power UV LED light by leveraging photocleavable ligand‐functionalized gold nanoparticles. Photocleavable protecting groups have been used to provide spatial and temporal control over the release of various chemicals for applications in biochemistry, biomedicine, and fluorescence activation.^[^ ^34^ ^]^ We are the first to report using photocleavable ligands, *o*‐nitrobenzyl thiol (NT) for this work, on gold nanoparticles (AuNPs) to control surface functionality for on‐demand ultralow power UV‐induced sintering. NT molecules are known to be photocleaved by 365 nm UV light.^[^ ^35^ ^]^ Upon exposure to UV light, NT ligands undergo desorption from the AuNP surface, as confirmed by our ^1^H NMR and FT‐IR studies. This leads to enhanced light absorption by reducing the filling factor of voids in the printed AuNPs layer, which increases the temperature of the layer and further facilitates ligand desorption. This positive feedback mechanism accelerates UV‐induced particle coalescence and fusion, creating conductive pathways.

We demonstrate that inkjet‐printed NT‐AuNP patterns on flexible polymer substrates can be sintered using a 365 nm UV LED with a peak power density of 280 mW cm^−2^, achieving an electrical conductivity of 7.0 × 10^6^ S m^−1^. This conductivity is comparable to that of thermally sintered samples, as well as laser and IPL sintered samples, despite our UV source having peak power densities 4–6 orders of magnitude lower.^[^ ^11^, ^13^, ^22^, ^23^, ^24^, ^25^, ^26^, ^27^, ^28^, ^29^, ^30^ ^]^ Low‐power UV‐induced sintering, in contrast to high‐power IPL sintering, is suitable for heat‐sensitive substrates and polymeric layers, as UV light has a penetration depth (<10 µm) up to 100 times shorter than IPL. Moreover, AuNPs are inherently more stable and less prone to oxidation than AgNPs, making the gold ink reported here well‐suited for ensuring long‐term stability and reliability, particularly in printed bioelectronics applications. Our method of incorporating *o*‐nitrobenzyl thiol ligands onto the AuNP surface can also be applied to other metallic nanoparticles, metal oxides, and quantum dots that can form metal‐sulfur bonds, enabling the development of various functional nanoinks for printed electronics.

## 2 Results and Discussion

### 2.1 Synthesis of Photocleavable Ligand‐Functionalized AuNPs

**Figure 1:**
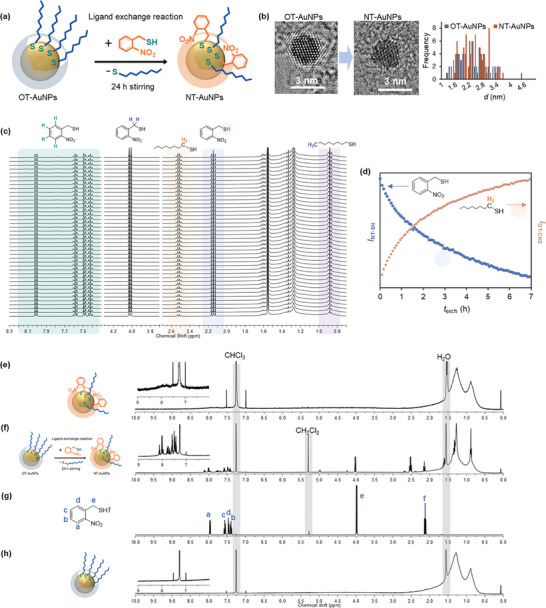
Photocleavable ligand (*o*‐nitrobenzyl thiol)‐functionalized gold nanoparticles (NT‐AuNPs). a) Schematic of the ligand exchange reaction between NT molecules and octanethiol (OT)‐AuNPs. b) (Left) TEM images of OT‐AuNPs and NT‐AuNPs and (Right) particle size distribution before and after the ligand exchange reaction. c) Ligand exchange reaction monitoring as a function of time (*t*_exch_) using ^1^H NMR. ^1^H NMR spectra were taken every 5 min for 7 h. d) The intensities of thiol proton of incoming NT molecules (*I*_NT‐SH_) and methylene protons of exiting OT ligands from the AuNP surface (*I*_OT‐CH2_) as a function of *t*_exch_. ^1^H NNR spectra of e) the purified NT‐AuNPs, f) the unpurified NT‐AuNPs after 24 h of the ligand exchange reaction, g) NT molecules, and h) OT‐AuNPs before the ligand exchange reaction. CDCl_3_ was used for all experiments.

Surface capping of AuNPs with photocleavable ligands such as NT is challenging due to the reduction of nitro groups in NT ligands to amino groups during synthesis using the Brust method. To incorporate NT ligands onto the surface of AuNPs, we employed a two‐step synthesis method: i) the synthesis of octanethiol (OT)‐functionalized AuNPs (OT‐AuNPs) and ii) ligand exchange reaction between NT molecules and the bound OT ligands (**Figure** **1**a). OT‐AuNPs with an average core diameter of 2.3 ± 0.6 nm were synthesized using the modified Brust method.^[^ ^11^ ^]^ NT molecules were synthesized following the previous protocol (Figure S1, Supporting Information),^[^ ^36^ ^]^ and the ligand exchange reaction was then carried out by mixing a dispersion of OT‐AuNPs in dichloromethane (DCM) with NT molecules in DCM at a 50:1 feed molar ratio of NT molecules to a single OT‐AuNP for 24 h and then separating and purifying AuNPs using a centrifuge. The average diameter of AuNPs (2.3 ± 0.6 nm) remained the same after the ligand exchange reaction, as confirmed by transmission electron microscopy (TEM) analysis (Figure 1b). This indicates that the conditions used for the ligand exchange reaction did not affect the particle size and did not induce particle coalescence.

^1^H NMR spectroscopy is used to probe the surface chemistry of MeNPs, including composition, binding interactions, and exchange dynamics. For larger particles (*d* > 2.0 nm), compared to ultrasmall metal clusters, resonance line broadening occurs due to ligand‐nanoparticle interactions, such as restricted molecular mobility, increased heterogeneity, and the paramagnetic effect of plasmonic nanoparticles, leading to faster relaxation.^[^ ^37^, ^38^, ^39^ ^]^ Despite this, NMR effectively monitors ligand exchange reactions. We conducted a time‐dependent ^1^H NMR study to track the exchange between NT molecules and bound OT ligands on the AuNP surface. Freely dissolved incoming NT ligands and exiting OT ligands were distinguished from the bound ligands by differences in their line widths and chemical shifts. Time‐dependent ^1^H NMR spectroscopy revealed that the peak intensity of the thiol proton of freely dissolved NT ligands (*I*_NT‐SH_) gradually decreased over the reaction time (*t*_exch_), while the peak intensity of the methylene protons of the exiting OT ligands (*I*_OT‐CH2_) increased, indicating the progress of the ligand exchange reaction (Figure 1c,d). After 24 h of the ligand exchange reaction, the final product was purified by centrifugation with ethanol three times to remove unbound ligands, as confirmed by ^1^H NMR spectra (Figure 1e–h). The broad peaks between 7.3 and 8.5 ppm, and between 0.5 and 2.25 ppm, correspond to the bound NT and OT ligands, respectively (Figure 1e). The ^1^H NMR spectrum of the purified sample showed that both OT and NT ligands remained on the AuNP surface after the ligand exchange reaction (Figure 1e). The successful incorporation of NT ligands onto the AuNP surface was also confirmed by FT‐IR spectroscopy, as evidenced by two nitro group peaks at 1521 and 1340 cm^−1^ (Figure S2, Supporting Information). The thermogravimetric analysis (TGA) result (Figure S3) shows that 10% of the total ligand population, corresponding to 14% of the sample weight, can be removed with UV exposure, indicating the ligand weight ratio of NT to OT ligands of ≈2.5:1. Given that thiols are generally strongly bound to all faces of AuNPs, we do not anticipate significant anisotropy in the surface distribution of the ligands.^[^ ^40^ ^]^

### 2.2 Photocleavage of NT Ligands from AuNPs

**Figure 2:**
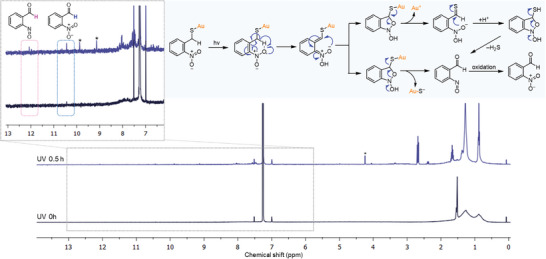
Photocleavage of NT ligands from the AuNP surface upon UV irradiation. ^1^H NMR spectra of NT‐AuNPs solutions in CDCl_3_ before and after 30 min of UV irradiation (*λ* = 365 nm) and (Inset) a scheme of proposed pathways and intermediates of photocleavage of NT ligands from the surface of AuNPs upon UV irradiation.

*o*‐Nitrobenzyl moiety is one of the most popular photocleavable protecting groups employed in the preparation of photo‐labile polymers.^[^ ^35^, ^41^ ^]^ The prior mechanistic studies of photolysis of *o*‐nitrobenzyl moiety on polymers revealed the production of *o*‐nitrosobenzaldehyde as a result of the UV‐induced cleavage of the *o*‐nitrobenzyl moiety.^[^ ^41^, ^42^, ^43^ ^]^ We performed ^1^H NMR studies to investigate whether this photolysis mechanism applies to the removal of NT ligands on the surface of AuNPs. The dispersion of NT‐AuNPs in CDCl_3_ (0.7 mL with 5 mg mL^−1^) in an NMR tube was irradiated under 282 mW cm^−2^ of UV light (365 nm) for ^1^H NMR analysis. The broad aromatic signals in the range of 7–8.5 ppm, corresponding to the bound NT ligands, were still detected in the 30‐min UV‐irradiated solution, indicating incomplete photoinduced removal of NT ligands from AuNPs (**Figure** **2**). After UV irradiation for 30 min, four new peaks appeared in the range of 9–13 ppm, indicating the formation of photolysis products. The peaks at 12.07 and 10.44 ppm are hypothesized to result from the formation of *o*‐nitrosobenzaldehyde and *o*‐nitrobenzaldehyde (a common product of *o*‐nitrobenzyl photolysis upon the oxidation of *o*‐nitrosobenzaldehyde), respectively.^[^ ^44^, ^45^ ^]^ These results are confirmed by our density functional theory (DFT) calculation of ^1^H NMR chemical shifts (Figure S4 and Table S1–S6, Supporting Information). The peaks at 9.88 and 9.13 ppm (denoted by asterisks in Figure 2) are likely due to the presence of intermediate species and secondary photolysis products arising from the photocleaved NT ligands and subsequent rearrangement in the reaction medium (Scheme S1, Supporting Information). The increase in the intensity of the peak at 2.68 ppm indicates the partial desorption of OT ligands due to the heat generated during UV exposure, which reached 75 °C after 5 min (Figure S5, Supporting Information).^[^ ^11^ ^]^ FT‐IR analysis on the NT‐AuNPs before and after UV irradiation for 30 min showed the disappearance of the peaks corresponding to two nitro groups at 1521 and 1340 cm^−1^, confirming the desorption of NT ligands (Figure S6, Supporting Information).

### 2.3 UV‐Induced Sintering and Electrical Performance

To demonstrate UV‐induced sintering of NT‐AuNPs, we formulated the nanoink consisting of 30 wt.% of NT‐AuNPs in 2‐butoxyethyl acetate for inkjet printing. The ink reservoir was stored in the dark to prevent any photodesorption that might occur under ambient light. The average core size (2.4 ± 0.7 nm) of NT‐AuNPs in the ink after 10 months remained unchanged compared to fresh NT‐AuNPs (2.3 ± 0.6 nm), demonstrating the long‐term stability of the ink (Figure S7, Supporting Information). A rectangular path (7 mm × 0.01 mm) was printed onto a poly(ethylene naphthalate) (PEN) substrate using a drop‐on‐demand inkjet printer (Dimatix, Fujifilm) with a nozzle temperature of 35 °C, substrate temperature of 60 °C and drop spacing of 10 µm. The electrical resistance of the printed NT‐AuNP pattern was measured using a two‐point probe method after exposure to a 365 nm UV light (282 mW cm^−1^).

**Figure 3:**
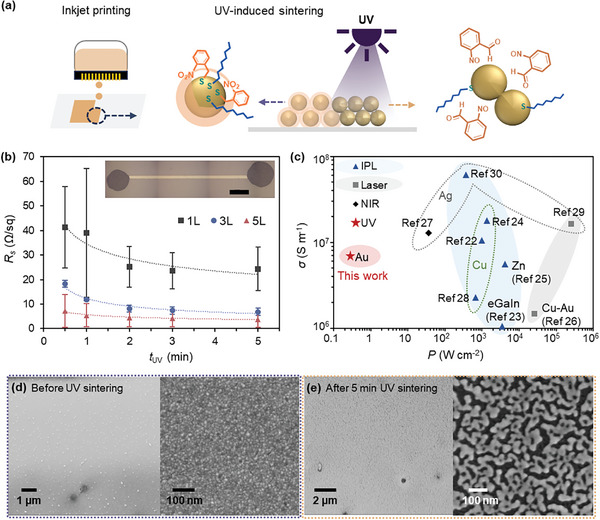
UV‐induced photonic sintering of the inkjet‐printed NT‐AuNPs on a flexible polymer substrate. a) Schematic description of inkjet printing the nanoink and UV‐induced NT ligand cleavage and nanoparticle sintering. b) Sheet resistance (*R*_s_) of inkjet‐printed NT‐AuNP conductive patterns with different printing layers on a polymer substrate as a function of UV irradiation time (*t*_UV_). (Inset) A photograph of the inkjet‐printed NT‐AuNP pattern with a single printed layer on a polymer substrate. The scale bar represents 1 mm. c) Electrical conductivity (*σ*) versus peak power density (*P*) for the comparison of ultralow power UV sintering (this work) with other photonic sintering results. Representative SEM images of inkjet‐printed NT‐AuNPs patterns d) before and e) after UV irradiation for 5 min.

The sheet resistance (*R_s_*) of printed NT‐AuNP patterns decreased with increasing UV exposure time (*t*_UV_) and with increasing the number of printed layers (**Figure** **3**). The electrical conductivity of a five‐layer printed NT‐AuNP pattern on a PEN substrate changed from nonconductive for untreated samples to 3.7 × 10^6^ S m^−1^ (9.1 × 10^5^ S m^−1^ for a single printed layer) after 30 s of UV irradiation and further increased to 7.0 × 10^6^ S m^−1^ (1.5 × 10^6^ S m^−1^ for a single printed layer) after 5 min of UV irradiation. This conductivity is comparable to that of laser and IPL‐sintered samples, despite our UV source having peak power densities 4–6 orders of magnitude lower (Figure 3c).^[^ ^22^, ^23^, ^24^, ^25^, ^26^, ^27^, ^28^, ^29^, ^30^ ^]^ It is also comparable to thermally sintered Ag patterns made with commercially available silver inks.^[^ ^13^ ^]^ This demonstrates the efficacy of the photocleavable ligands for ultralow power UV‐induced AuNP fusion. Scanning electron microscopy (SEM) images reveal that the surface morphology of the inkjet‐printed NT‐AuNP pattern changes after UV sintering, forming continuous films of interfused NPs (Figure 3d,e). TEM images of NT‐AuNPs exposed to UV irradiation for varying time intervals of 0, 0.5, 1, 5, and 30 min further confirmed the UV‐induced increase in nanoparticle size and the merging of nanoparticles (Figure S8, Supporting Information). We note that the inkjet‐printed OT‐AuNPs pattern remained insulating even after 10 min of UV irradiation.

### 2.4 Photothermal Effect

**Figure 4:**
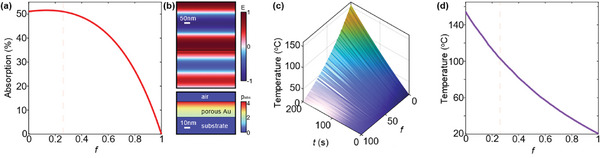
Simulation results of the photothermal effect in the nanoparticle layer. a) Optical absorption at 365 nm with different values of the filling factor (*f*) of air. b) Spatial distributions of the electric field (*E*) and the absorption power density (*P_abs_*) along the polarization direction. c) The time‐dependent temperature of the nanoparticle layer as a function of *f*. d) The temperature after 200 s of UV illumination as a function of *f*. The temperature is the spatial average over the layer.

To investigate the photothermal effect on nanoparticle sintering induced by photodesorption of NT ligands from the AuNP surface, we performed numerical simulations using Maxwell's equations coupled with the heat equation. Since the average core diameter (2.3 ± 0.6 nm) of NT‐AuNPs is much smaller than the optical wavelength (365 nm in this work), we modeled the printed layer, composed of closely packed NT‐AuNPs within the printed pattern, as a porous medium and applied effective medium theory for electromagnetic and heat conduction simulations. The parameter, *f*, represents the volume fraction (filling factor) of air within the closely packed nanoparticles. **Figure** **4**a shows the simulated optical absorption as a function of *f* when a linear polarized light (365 nm) is applied. The interparticle distance is expected to decrease as NT ligands are cleaved from the surface of AuNPs upon exposure to UV light, leading to a reduction in the value of *f* and an increase in optical absorption. This is corroborated by TGA analysis showing the weight loss (≈10%) of the sample after UV irradiation due to the desorption of NT ligands (Figure S3, Supporting Information). Figure 4b shows the spatial distribution of the electric field (*E*) along the polarization direction, revealing significant attenuation due to the power absorption (*P_abs_*) by the layer of AuNPs. For these simulations, we set *f* = 26%, corresponding to *f* for hexagonal close‐packed solid spheres of identical size. We then coupled the absorption power into the heat equation to calculate the temperature evolution within the nanoparticle layer. Figure 4c,d shows that the layer of AuNPs at smaller *f* is heated to higher temperatures due to increased absorption, facilitating further desorption of the ligands from the surface of AuNPs. This positive feedback mechanism accelerates the UV sintering of AuNPs for achieving high conductive pathways.

## 3 Conclusion

In summary, we synthesized photocleavable ligands (*o*‐nitrobenzyl thiol) – functionalized gold nanoparticles via the ligand exchange reaction. The results demonstrate that *o*‐nitrobenzyl thiol can be effectively removed from the surface of AuNPs using an ultralow power UV LED source. UV‐induced desorption of NT ligands leads to light absorption enhancement by reducing the filling factor of voids in the printed layer. This drives the system to a higher temperature, facilitating further desorption of NT ligands from AuNPs and then mediating particle fusion. This positive feedback mechanism accelerates UV sintering of AuNPs, allowing for particle fusion and achieving an electrical conductivity of 7.0 × 10^6^ S m^−1^, with an energy input that is several orders of magnitude lower than that required by other photonic sintering methods such as laser and IPL. Our findings provide a strategy for 3D printing metallic patterns using photocleavable surface ligands on AuNPs, enabling on‐demand nanoparticle sintering with low‐power UV light sources.

## 4 Experimental Section

### Materials

All materials were purchased from Aldrich and used as received. Poly(ethylene naphthalate) substrate (75 µm thickness) films were supplied by GTS Flexible Materials LTD.

### Synthesis of o‐nitrobenzyl Thiol

*o*‐Nitrobenzyl thiol was synthesized via two steps by following the previous protocol^[^ ^36^ ^]^ and confirmed by ^1^H NMR (Figure S1, Supporting Information). First, a mixture of *o*‐nitrobenzyl bromide (6.0 g, 27.8 mmol) and thiourea (2.43 g, 31.9 mmol) in THF (100 mL) was stirred overnight at room temperature. The white precipitate (Compound 1, Figure S1, Supporting Information) was formed after the reaction and was filtered and washed with ethyl acetate. The white solid was dried under vacuum (7.7 g, yield: 94%). Then, 4.29 g of Compound 1 (14.7 mmol) was suspended in a mixture of dichloromethane (80 mL) and deionized water (60 mL), and 11.2 g of sodium metabisulfite (58.7 mmol) was added. The suspension was refluxed for 4 h and then cooled to room temperature. The organic phase was collected, dried with Na_2_SO_4_, and concentrated to obtain the product of 1.92 g (yield: 77%). Compound 1: ^1^H NMR (400 MHz, DMSO‐*d*_6_, δ) 9.17 (br, 4H), 8.15–8.12 (dd, *J* = 4 Hz, *J* = 8 Hz, 1H), 7.83–7.73 (m, 2H), 7.68–7.63 (m, 1H), 4.77 (s, 2H); *o*‐nitrobenzyl thiol: ^1^H NMR (400 MHz, CDCl_3_, δ) 7.98–7.95 (dd, *J* = 4 Hz, *J* = 8 Hz, 1H), 7.59–7.55 (m, 1H), 7.47–7.37 (m, 2H), 3.97 (d, *J* = 12 Hz, 2H), 2.12 (t, *J* = 8 Hz, 1H)

### Synthesis of Octanethiol Functionalized Gold Nanoparticles (OT‐AuNPs)

The synthesis of OT‐AuNPs was performed by the modified Brust method.^[^ ^11^ ^]^ Gold (III) chloride trihydrate (0.0104 mol, HAuCl_4_∙3H_2_O) in the aqueous phase (50 mL) was transferred by a phase transfer agent, tetraoctylammonium bromide (0.021 mol, (C_8_H_17_)_4_NBr), into the octanethiol (0.0035 mol) solution in toluene (100 mL) using 3:1 of AuCl_4_^−^/thiol molar ratio. Aqueous sodium borohydride (0.104 mol, NaBH_4_) was then added dropwise while stirring, and the mixture was stirred vigorously for 3 h under ambient conditions. The dark, violet‐colored organic phase was separated from the aqueous phase, rotary evaporated to 15 mL, and diluted to 300 mL of ethanol. The product was washed five times with ethanol separated by centrifugation and dried under vacuum.

### Ligand Exchange Reaction

A Ligand exchange reaction was carried out by adding the solution of NT ligands (2%) in dichloromethane to a flask containing the solution of OT‐AuNPs (28 mg mL^−1^ in DCM). A 50:1 feed molar ratio of NT ligands to a single OT‐AuNP was used for the ligand exchange reaction. The mixture was stirred at room temperature in the dark for 1 day and the particles were purified with ethanol three times using a centrifuge at 10 °C and dried under vacuum.

### Characterization

^1^H NMR was performed on a Bruker AV III 400HD NMR spectrometer. Experiments were carried out to confirm the structure of NT ligands, monitor the ligand exchange reaction, and study the photocleavage of NT ligands from AuNPs after UV irradiation. Standard ^1^H NMR for ligand and nanoparticle analysis was carried out using a zg30 pulse sequence with the transmitter frequency set to 6.175 ppm, a spectral width of 20.55 ppm, and 64k points. Spectra were run with either 32 or 64 scans depending on the signal‐to‐noise required. For the ligand exchange reaction, first, an inversion recovery experiment was carried out on the NT ligand. From this the longest T1 was determined to be 7 s. The NMR sample for the ligand exchange reaction was made up in the lab and transported to the NMR leading to 20 min delay between the sample being made and the analysis starting. Once loaded in the NMR, the spectra were acquired every 5 min using a zg pulse sequence with the transmitter frequency set to 5.5 ppm, a spectral width of 11 ppm, and 64 k points. The relaxation delay (d1) was set to 28 s and the AQ was 7.45 s (due to the sweep width and number of points used) leading to an interscan delay of 35.45 s (greater than 5 × T1 calculated from the inversion experiment). In total 84 experiments were run. The data was imported into Mnova using the directory spectra stack script and analyzed using the arrayed data analysis tools. FT‐IR analysis in attenuated total reflection (ATR) mode was carried out using PerkinElmer Frontier FT‐IR spectrometer. Baseline correction was not performed to prevent inaccurate interpretation of results. For TGA, PerkinElmer TGA4000 was used, and the sample was heated from 50 to 600 °C at a rate of 10 °C min^−1^ in air. TEM studies on the size of AuNPs were performed on JEOL 2100F FEG TEM operating at an accelerating voltage of 200 kV and equipped with a Gatan K3‐IS camera. ImageJ software was used to analyze the mean core diameter of AuNPs. SEM was carried out using Zeiss Crossbeam 550 to characterize the surface morphology of the inkjet‐printed gold patterns on PEN substrates before and after UV sintering. The optical microscope images were acquired using optical microscopy (Nikon Eclipse LV100ND). The film thicknesses of the inkjet‐printed nanoparticle layers on both glass and polymer substrates were determined through surface topography measurements of both single‐layer and five‐layer printed samples. Surface topography was measured using a Zygo Nexview NX2, a coherence scanning interferometer‐based instrument. The system featured a 5.5× magnification objective lens and a 0.5× magnification tube lens, providing a total field of view of 3.15 mm × 3.15 mm. The thickness of the sample was determined relative to the substrate along its entire length. The presence of a surface form due to the polymer substrate could introduce errors in the thickness measurement over large areas. To minimize this error, the stitching feature‐commonly used to combine multiple measurements for an extended field of view – was not employed. Instead, two distinct measurements were conducted for each sample to capture the full length. For each surface topography, the mean profile was extracted through averaging the profiles along the y‐axis (Figure S9, Supporting Information). The thickness values were determined by processing the surface topography data using MountainsMap software, following the guidelines outlined in ISO 16610–20^[^ ^46^ ^]^ and ISO 5436‐1.^[^ ^47^ ^]^ Each measurement was repeated three times to ensure repeatability, and the average values obtained from the various measurement areas and repeats were reported as layer thickness.

### Computational Methods

All structural optimization calculations were performed using Becke's three‐parameter hybrid exchange functionals, the Lee‐Yang‐Paar correlation functional, and Grimme's D3 dispersion force correction method (B3LYP‐D3)^[^ ^48^, ^49^ ^]^ with the 6–31 + G** basis set implemented in the Gaussian16 Revision B.013^[^ ^50^ ^]^ suite of programs with default thresholds and algorithms. The stationary points were optimized without any symmetry assumptions and were characterized by frequency analysis at the same level of theory (the number of imaginary frequencies, NIMAG, was 0). Structural optimization and NMR calculations in the cavity reaction field in the solvent (CHCl_3_) were carried out using the SCRF method based on the polarizable continuum model. The optimized structures and the Cartesian coordinates are shown in Figure S4 and Tables S1–S6 (Supporting Information).

### Ink Formulation and Inkjet Printing

The ink containing NT‐AuNPs was formulated with 30 wt.% of NT‐AuNPs in 2‐butoxyethyl acetate. The particles were dispersed using a bath sonicator for 20 min and stored in the dark in a laboratory refrigerator (4 °C) before use. The ink was deposited using a Fujifilm Dimatix Materials Printer (DMP‐2850) with a 2.4 pL cartridge (Samba cartridge). The nozzle temperature of 35 °C was used to generate a stable droplet. For electrical properties measurement, the ink was printed using one nozzle for high‐quality printing, drop spacing of 10 µm, jetting frequency of 1 kHz, and substrate temperature of 60 °C. The photonic sintering was performed using an array of UV LED (365 nm, Phoseon Technology, FireFly UV lamp). The 5 mm distance between the printed pattern and the UV lamp was kept for all UV sintering experiments.

### Electrical Properties Measurement

For sheet resistance measurement, a printed sample on a PEN substrate was sintered using a UV light (365 nm, 282 mW cm^−2^). The power density of a UV source was measured using a photodiode power sensor (S120VC, Thorlabs PM100d). Electrical contacts were made using a conductive silver paint (Agar quick‐drying silver paint, 25% of silver powder (<100 nm) suspended in methyl isobutylketone). The resistance of the printed sample was measured using a two‐probe method (digital multimeter, UNI‐T UT50B). The sheet resistance (*R_s_*) was calculated from the equation *R_s_* = *R* × (*W*/*L*), where *R*, *W*, and *L* are the resistance, width, and length of a sample, respectively. The electrical conductivity (*σ*) was calculated using the following equation: *σ = 1/(R_s_* × *t)*, where *t* is the thickness of a printed layer. *L* and *W* were measured using an optical microscope, and *t* of the single‐layer and five‐layer samples on PEN substrates were measured to be 26 ± 0.27 nm and 37 ± 0.58 nm, respectively, using the optical profiler (Figure S9, Supporting Information).

### Optical and Heat Simulation

A finite‐element‐method (FEM) solver was used for the simulations, which coupled the Maxwell's equation with the heat equation. The absorption power density was calculated using the equation $p_{abs}=-\frac{1}{2}Re\left(E\times H\right)$, which worked as the heat source in the heat equation. A linearly polarized light was set as the light source, with a wavelength of 365 nm and a power density of 280 mW cm^−2^. Since the average core diameter of the nanoparticle (2.3 ± 0.6 nm) was much smaller than the optical wavelength, the layer of NT‐AuNPs was treated as a bulk medium, with the complex refractive indexed derived using effective medium theory. For the simulation, the volume contribution of the ligand was neglected. For a medium with a fixed filling factor, the effective permittivity was calculated using the Maxwell–Garnett approximation.^[^ ^51^, ^52^ ^]^ Similarly, the effective heat conductivity of the medium was determined using the Maxwell‐Eucken equation.^[^ ^53^ ^]^ The values of the complex refractive index and heat conductivity at different filling factors were plotted in Figure S10. The remaining parameters, including mass density and heat capacitance, are linear combinations of the constituent materials. The refractive index of gold used in the simulations was obtained from the literature.^[^ ^54^ ^]^ Due to the thin layer of a single printed NT‐AuNPs (average 26 nm), the minimum size of the mesh was set to 2 nm, to ensure adequate resolution along the vertical direction. Periodic boundary condition was used along the transverse direction, while thermal isolation boundary condition was used for the top and bottom planes 0.75 and 1.5 µm away from the absorbing layer, respectively.

## Conflict of Interest

The authors declare no conflict of interest.

## Supporting information



Supporting Information

## Data Availability

The data that support the findings of this study are available in the supplementary material of this article.
