# Supplementary material for: On‐Demand Sintering of Gold Nanoparticles via Controlled Removal of o‐Nitrobenzyl Thiol Ligands Under Record‐Low Power for Conductive Patterns
Source: Adv Sci (Weinh). 2025 Jan 31;12(12):2415496. doi: 10.1002/advs.202415496 (PMC11948040; doi:10.1002/advs.202415496)
Supplement: Supplementary file 1 — Supporting Information [file ADVS-12-2415496-s001.docx]

Supporting Information

On-demand Sintering of Gold Nanoparticles via Controlled Removal of *o*-Nitrobenzyl Thiol Ligands under Record-low Power for Conductive Patterns

Jisun Im,^1,2,*^ Charles Heaton,^1^ Nur R. E. Putri,^1^ Changxu Liu,^3^ Junichi Usuba,^4^ Kevin Butler,^5^ Michael Fay,^6^ Grace G. D. Han,^7^ Helia Hooshmand,^8^ Adam Thompson,^8^ Ricky Wildman,^1^ Richard Hague,^1^ Lyudmila Turyanska^1^ and Christopher Tuck^1,*^

^1^Centre for Additive Manufacturing, Faculty of Engineering, University of Nottingham, Nottingham, NG7 2RD, UK

^2^School of Engineering, University of Warwick, Coventry, CV4 7AL, UK

^3^Centre for Metamaterial Research and Innovation, Department of Engineering, University of Exeter, Exeter, EX4 4PY, UK

^4^Research Center for Net Zero Carbon Society, Institute of Innovation for Future Society, Nagoya University, Nagoya, Japan

^5^School of Chemistry, University of Nottingham, Nottingham, NG7 2RD, UK

^6^Nanoscale and Microscale Research Centre, University of Nottingham, Nottingham, NG7 2RD, UK

^7^Department of Chemistry, Brandeis University, Waltham, MA 02453, USA

^8^Manufacturing Metrology Team, Faculty of Engineering, University of Nottingham, Nottingham, NG7 2RD, UK

**Table of Contents**

**Figure S1.** (a) Schematic of the synthesis of *o*-nitrobenzyl thiol. ^1^H NMR (400 MHz) spectra of (b) compound 1 in DMSO-d_6_ and (c) *o*-nitrobenzyl thiol in CDCl_3_. * annotating a residual solvent peak. …………………………………………………………………………….……..4

**Figure S2.** FT-IR spectra of OT-AuNPs before ligand exchange reaction and NT-AuNPs after ligand exchange reaction. Two peaks for a nitro (-NO_2_) group appeared at 1521 cm^-1^ and 1340 cm^-1^ after ligand exchange reaction and purification, confirming the successful incorporation of NT ligands on the surface of AuNPs. FT-IR measurement was carried out in ATR (Attenuated Total Reflection) mode at ambient room conditions. Baseline correction was not performed. ………………………………………………………………………………..........5

**Figure S3.** TGA analysis of NT-AuNPs before and after 30 minutes of UV irradiation. The weight loss is due to the desorption of NT ligands……………………………….....................5

**Scheme S1.** The proposed mechanisms of the photocleavage of NT ligands from AuNPs……6

**Figure S4.** Optimized structures of the proposed intermediates and products (A-F) and the predicted ^1^H NMR chemical shifts (ppm) for each structure (calculated at the B3LYP-D3/6-31G** level of theory incorporating in CHCl_3_ as the solvent). ……………………..................6

**Table S1.** Cartesian coordinates (Å) of the optimized geometry for A’. ………………………7

**Table S2.** Cartesian coordinates (Å) of the optimized geometry for B. …………………….....7

**Table S3.** Cartesian coordinates (Å) of the optimized geometry for C. …………………….....8

**Table S4.** Cartesian coordinates (Å) of the optimized geometry for D. ……………….............8

**Table S5.** Cartesian coordinates (Å) of the optimized geometry for E. ……………………….9

**Table S6.** Cartesian coordinates (Å) of the optimized geometry for F. ……………………….9

**Figure S5.** Substrate heating due to UV light. The distance between the UV LED source and the PEN substrate was kept at 5 mm. …………………………………………………………10

**Figure S6.** FT-IR spectra of NT-AuNPs before and after UV irradiation (365 nm, 282 mW cm^-2^) for 30 minutes. Two peaks at 1521 cm^-1^ and 1340 cm^-1^ for a nitro (-NO_2_) group disappeared after UV irradiation, confirming the desorption of NT ligands. FT-IR measurement was carried out in ATR mode at ambient room conditions. Baseline correction was not performed to prevent inaccurate interpretation of results. .........................................................................................10

**Figure S7.** Particle size monitoring. (a) the average core diameters and (b) particle size distributions of OT-AuNPs, NT-AuNPs after ligand exchange reaction, and (c) NT ink stored for 10 months. …………………………………………………………………………...........11

**Figure S8.** Representative TEM images of the areas with high density of NT-AuNPs exposed to UV irradiation for 0, 0.5, 1, 5, and 30 minutes. .....................................................................11

**Figure S9.** The film thicknesses of inkjet printed NT-AuNP patterns on poly(ethylene naphthalate) (PEN) and glass substrates measured using the optical profiler. Surface topography and thicknesses of (a) a single-layer printed NT-AuNP pattern on a PEN substrate and (b) a five-layer printed NT-AuNP pattern on a PEN substrate. (c) The thickness profile of a single-layer printed NT-AuNP pattern on a glass substrate. …………..……………………………12

**Figure S10.** (a) The complex refractive index and (b) heat conductivity at different filling factor (*f*) of air in the nanoparticle layer. …………………………………………………….............12


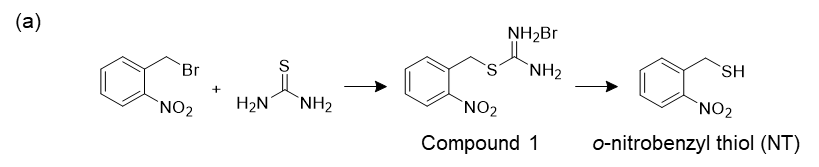

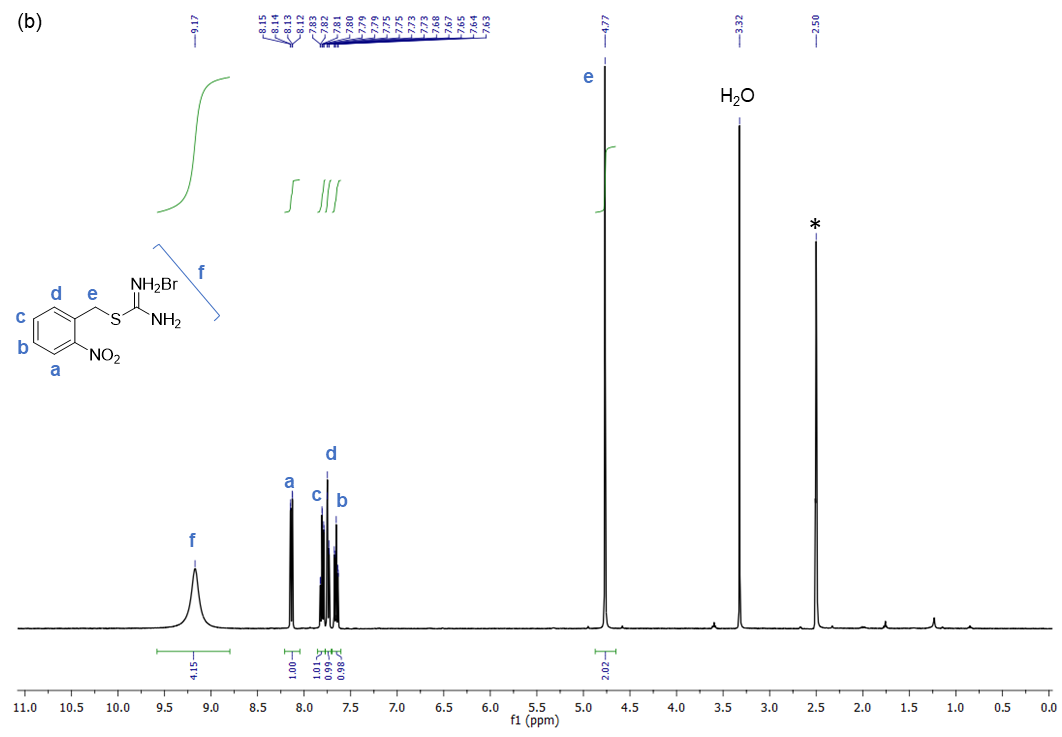

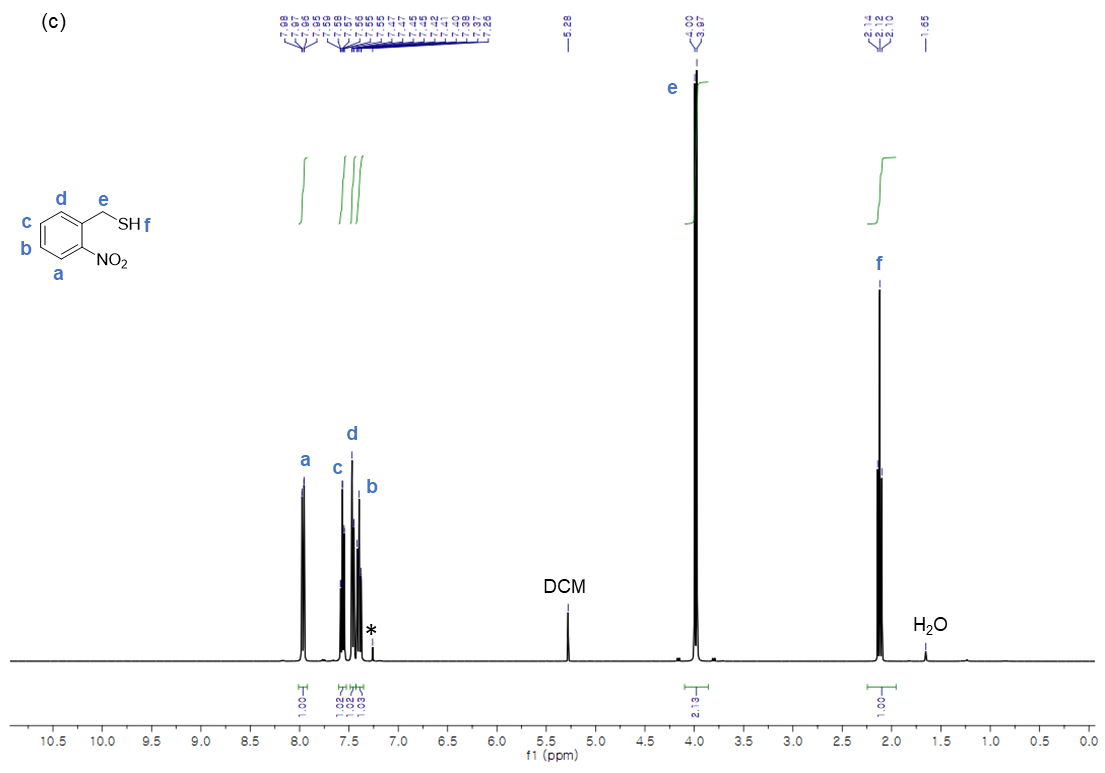


**Figure S1.** (a) Schematic of the synthesis of *o*-nitrobenzyl thiol. ^1^H NMR (400 MHz) spectra of (b) compound 1 in DMSO-d_6_ and (c) *o*-nitrobenzyl thiol in CDCl_3_. * annotating a residual solvent peak.


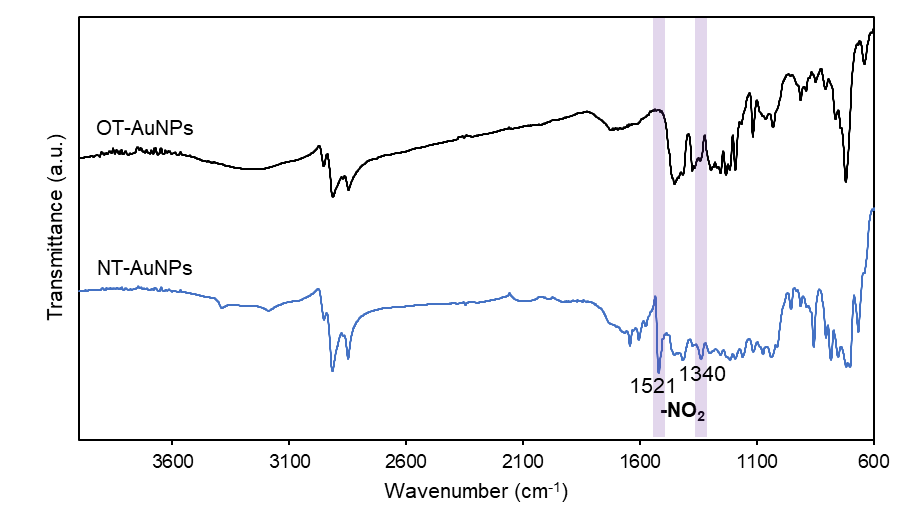


**Figure S2**. FT-IR spectra of OT-AuNPs before ligand exchange reaction and NT-AuNPs after ligand exchange reaction. Two peaks for a nitro (-NO_2_) group appeared at 1521 cm^-1^ and 1340 cm^-1^ after ligand exchange reaction and purification, confirming the successful incorporation of NT ligands on the surface of AuNPs. FT-IR measurement was carried out in ATR (Attenuated Total Reflection) mode at ambient room conditions. Baseline correction was not performed.


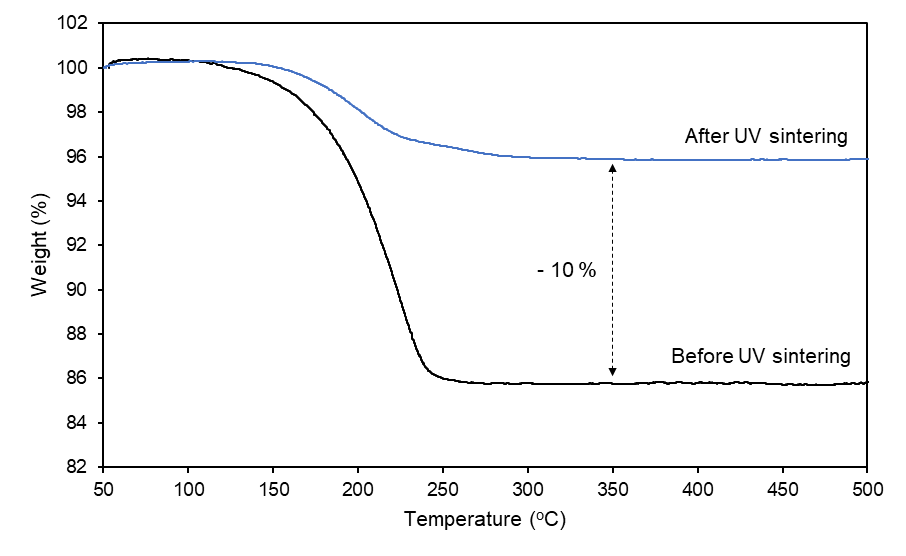
**Figure S3**. TGA analysis of NT-AuNPs before and after 30 minutes of UV irradiation. The weight loss is due to the desorption of NT ligands.

**Scheme S1**. The proposed mechanisms of the photocleavage of NT ligands from AuNPs.


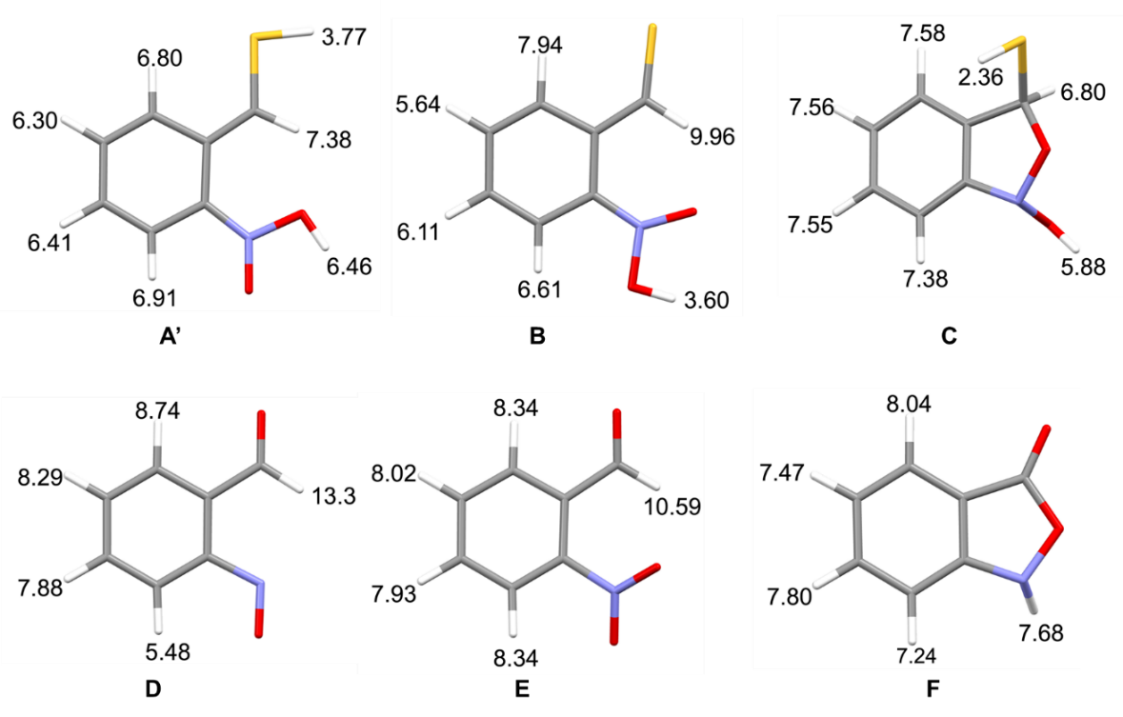
**Figure S4**. Optimized structures of the proposed intermediates and products (A-F) and the predicted ^1^H NMR chemical shifts (ppm) for each structure (calculated at the B3LYP-D3/6-31G** level of theory incorporating in CHCl_3_ as the solvent).

**Table S1.** Cartesian coordinates (Å) of the optimized geometry for A’.

C -1.90575 1.11335 0.00001

H -2.96500 0.89600 -0.00002

C -1.43499 2.38429 -0.00011

H -2.12938 3.21773 -0.00025

C -0.01872 2.63505 -0.00003

H 0.34454 3.65734 0.00002

C 0.85739 1.60241 0.00008

H 1.92172 1.81221 0.00034

C 0.45105 0.20414 0.00011

C -1.00737 -0.01747 0.00006

C 1.40750 -0.77707 0.00022

H 1.15103 -1.82160 0.00030

S 3.13955 -0.44240 -0.00018

H 3.52432 -1.73085 0.00168

N -1.62072 -1.20508 0.00003

O -2.85662 -1.44129 0.00015

O -0.80997 -2.35137 -0.00028

H -1.49693 -3.04365 -0.00032

**Table S2.** Cartesian coordinates (Å) of the optimized geometry for B.

H -1.99887 1.77761 0.00027

C -0.92695 1.60644 0.00017

C 1.82732 1.10892 -0.00026

C -0.52352 0.22865 -0.00013

C -0.05128 2.65327 0.00022

C 1.35391 2.38684 -0.00007

C 0.92479 -0.01864 -0.00018

H -0.40989 3.67791 0.00040

H 2.05980 3.21317 -0.00014

H 2.89057 0.91449 -0.00050

C -1.49155 -0.79091 -0.00040

H -1.09745 -1.79942 -0.00100

S -3.19482 -0.63027 0.00009

N 1.44049 -1.24619 -0.00002

O 2.87707 -1.32534 0.00025

H 2.95936 -2.29453 0.00021

O 0.86716 -2.39078 0.00017

**Table S3.** Cartesian coordinates (Å) of the optimized geometry for C

H -0.11721 -2.54521 0.91016

C -0.69454 -1.71485 0.51741

C -2.18976 0.45544 -0.52986

C -0.09350 -0.48883 0.26229

C -2.05823 -1.85010 0.23929

C -2.79614 -0.77800 -0.27673

C -0.83472 0.56386 -0.25397

H -2.54976 -2.80031 0.42056

H -3.85288 -0.90626 -0.48707

H -2.74828 1.29646 -0.92431

C 1.31314 -0.00198 0.44651

H 1.57851 0.13955 1.50099

N -0.02812 1.74439 -0.43072

O 1.32032 1.25794 -0.26467

S 2.63196 -1.07761 -0.21713

H 2.06376 -1.25775 -1.42637

O -0.33176 2.56058 0.72118

H -0.07464 3.44319 0.41348

**Table S4.** Cartesian coordinates (Å) of the optimized geometry for D

H -2.26907 1.38046 -0.00014

C -1.19605 1.22580 -0.00008

C 1.57170 0.77555 0.00008

C -0.71611 -0.08861 -0.00004

C -0.30805 2.29697 -0.00005

C 1.07670 2.07246 0.00005

C 0.67810 -0.30265 0.00002

H -0.68837 3.31330 -0.00009

H 1.76107 2.91448 0.00011

H 2.63476 0.56553 0.00017

N 1.12004 -1.67101 0.00007

O 2.33382 -1.82136 -0.00011

C -1.69180 -1.21453 -0.00001

H -1.26096 -2.22822 -0.00002

O -2.89689 -1.03344 0.00007

**Table S5.** Cartesian coordinates (Å) of the optimized geometry for E H 2.73021 0.75912 0.04727

C 1.92933 0.02947 0.00999

C -0.17162 -1.80523 -0.03217

C 0.61531 0.51159 0.04672

C 2.19721 -1.33508 -0.05613

C 1.14630 -2.25411 -0.07034

C -0.42148 -0.43690 0.01156

H 3.22411 -1.68302 -0.08918

H 1.34823 -3.31858 -0.11707

H -1.00647 -2.49344 -0.05380

N -1.82990 -0.02124 -0.03500

O -2.08648 1.08918 -0.50606

O -2.67487 -0.81305 0.37884

C 0.43629 1.98791 0.22092

H -0.54278 2.33101 0.58476

O 1.34484 2.76984 0.01343

**Table S6.** Cartesian coordinates (Å) of the optimized geometry for F.

H -0.49534 -2.53563 -0.01402

C -0.81140 -1.49809 -0.01161

C -1.59133 1.24621 0.00084

C 0.13529 -0.47112 -0.02221

C -2.15366 -1.13948 0.00865

C -2.53019 0.21773 0.02032

C -0.24707 0.86936 -0.02205

H -2.92137 -1.90536 0.01690

H -3.58527 0.47159 0.04177

H -1.89082 2.28815 0.00162

C 1.59288 -0.50921 0.00711

O 2.39271 -1.41727 0.01905

N 0.88846 1.68814 -0.11725

H 0.98823 2.38655 0.61791

O 2.02207 0.81544 0.01473


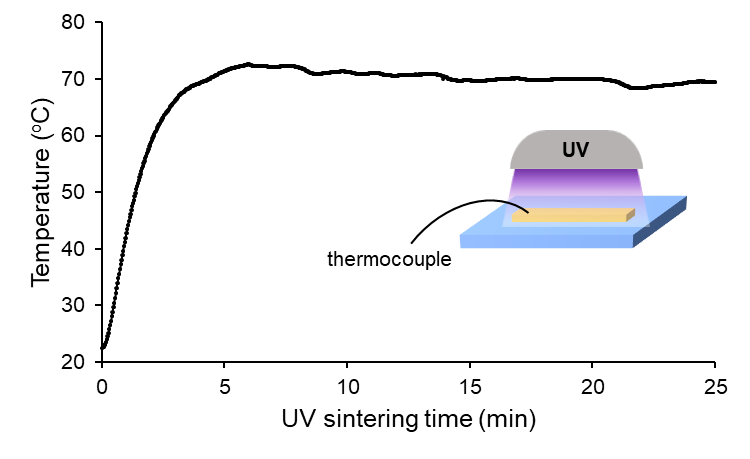


**Figure S5.** Substrate heating due to UV light. The distance between the UV LED source and the PEN substrate was kept at 5 mm.


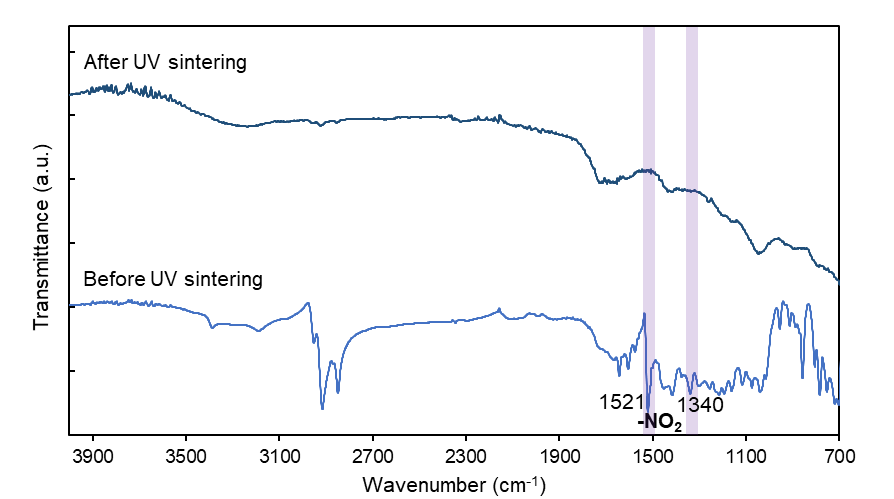


**Figure S6**. FT-IR spectra of NT-AuNPs before and after UV irradiation (365 nm, 282 mW cm^-2^) for 30 minutes. Two peaks at 1521 cm^-1^ and 1340 cm^-1^ for a nitro (-NO_2_) group disappeared after UV irradiation, confirming the desorption of NT ligands. FT-IR measurement was carried out in ATR mode at ambient room conditions. Baseline correction was not performed to prevent inaccurate interpretation of results.


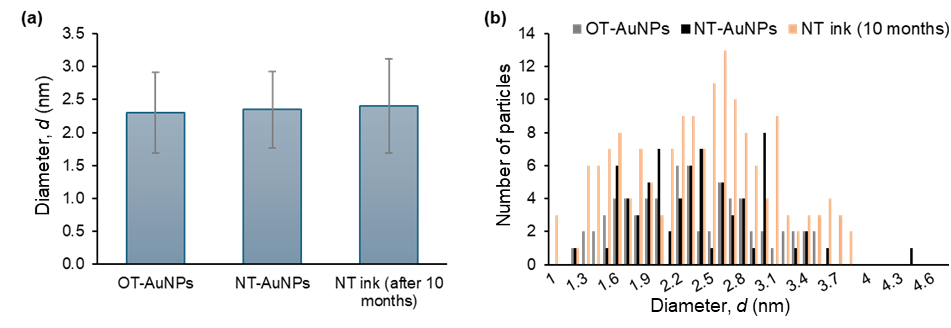


**Figure S7.** Particle size monitoring. (a) the average core diameters and (b) particle size distributions of OT-AuNPs, NT-AuNPs after ligand exchange reaction, and NT-AuNP ink stored for 10 months.

**
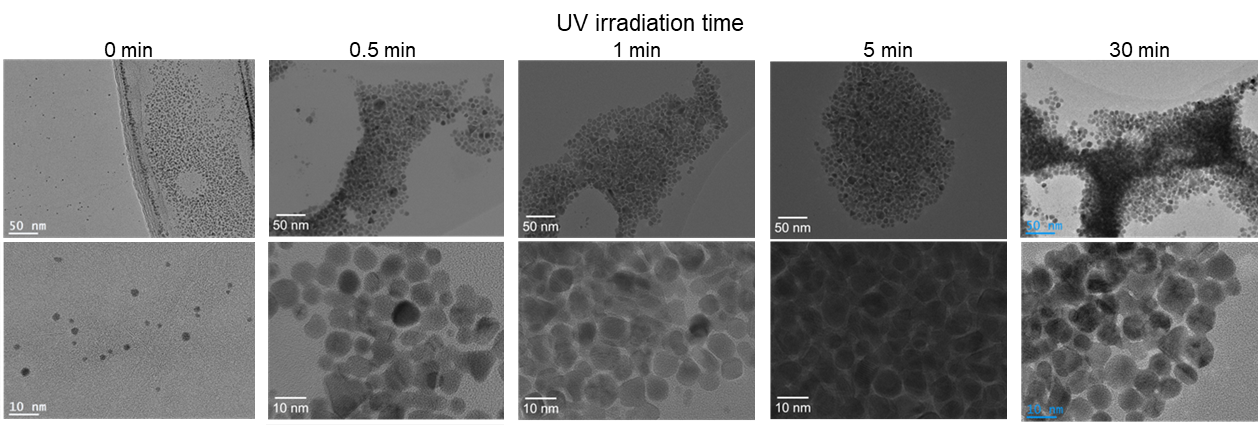
**

**Figure S8**. Representative TEM images of the areas with high density of NT-AuNPs exposed to UV irradiation for 0, 0.5, 1, 5, and 30 minutes.


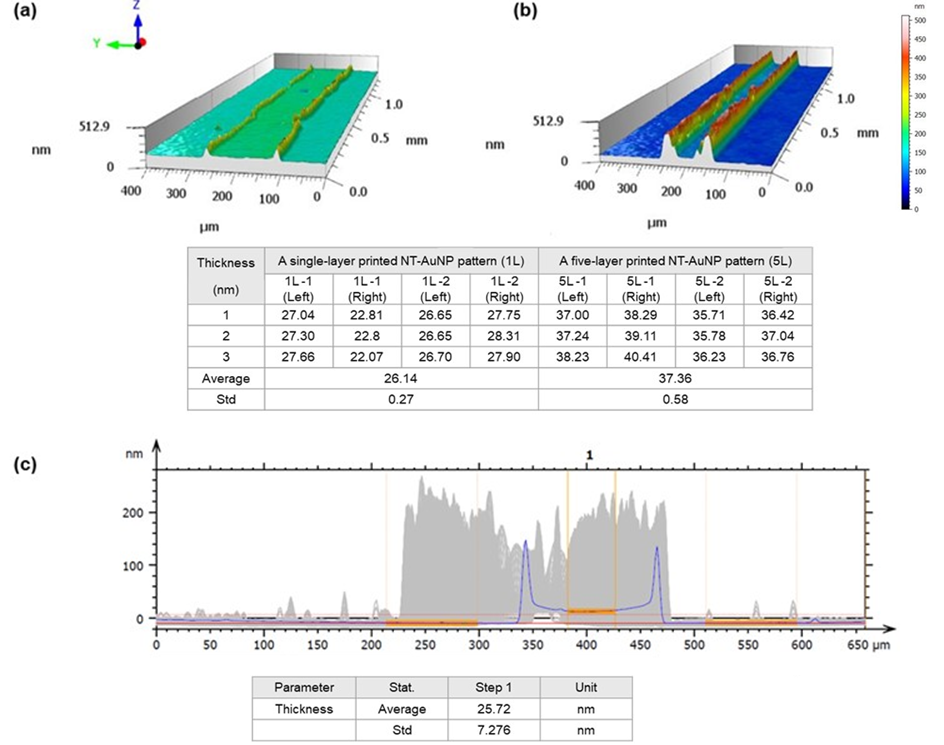


**Figure S9.** The film thicknesses of inkjet-printed NT-AuNP patterns on poly(ethylene naphthalate) (PEN) and glass substrates measured using the optical profiler. Surface topography and thicknesses of (a) a single-layer printed NT-AuNP pattern on a PEN substrate and (b) a five-layer printed NT-AuNP pattern on a PEN substrate. (c) The thickness profile of a single-layer printed NT-AuNP pattern on a glass substrate.


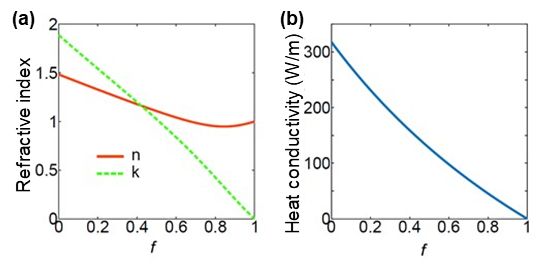


**Figure S10**. (a) The complex refractive index and (b) heat conductivity at different filling factor (*f*) of air in the nanoparticle layer.
